# (*S*)-2-(2-Pyrrolidinio)-1*H*-benzimidazol-3-ium dichloride monohydrate

**DOI:** 10.1107/S1600536809019084

**Published:** 2009-05-23

**Authors:** Dai Jing

**Affiliations:** a Ordered Matter Science Research Center, College of Chemistry and Chemical Engineering, Southeast University, Nanjing 210096, People’s Republic of China

## Abstract

In the title compound, C_11_H_15_N_3_^2+^·2Cl^−^·H_2_O, one N atom of the imidazole ring and the N atom of the pyrrolidine ring are protonated. The crystal structure is stabilized by aromatic π–π inter­actions between the benzene rings of neighbouring benzimidazole systems [centroid–centroid duistance = 3.712 (2) Å]. The crystal structure is further stabilized by inter­molecular N—H⋯Cl, O—H⋯Cl and N—H⋯O hydrogen bonds.

## Related literature

For proline derivatives, see: Fu *et al.* (2007 ▶); Aminabhavi *et al.* (1986 ▶). For related structures, see: Dai & Fu (2008*a* ▶,*b* ▶); Fu & Ye (2007 ▶).
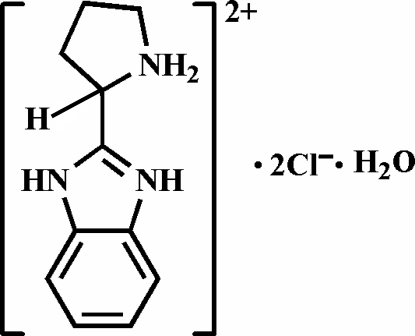


## Experimental

### 

#### Crystal data

C_11_H_15_N_3_^2+^·2Cl^−^·H_2_O
*M* *_r_* = 278.18
Triclinic, 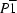

*a* = 7.493 (2) Å
*b* = 9.739 (2) Å
*c* = 9.937 (2) Å
α = 99.23 (3)°
β = 95.73 (3)°
γ = 106.27 (3)°
*V* = 679.0 (3) Å^3^
*Z* = 2
Mo *K*α radiation
μ = 0.47 mm^−1^
*T* = 293 K
0.35 × 0.30 × 0.15 mm

#### Data collection

Rigaku Mercury2 diffractometer
Absorption correction: multi-scan (*CrystalClear*; Rigaku, 2005 ▶) *T*_min_ = 0.959, *T*_max_ = 0.982 (expected range = 0.911–0.932)
7119 measured reflections
3108 independent reflections
2310 reflections with *I* > 2σ(*I*)
*R*_int_ = 0.037

#### Refinement

*R*[*F* ^2^ > 2σ(*F* ^2^)] = 0.050
*wR*(*F* ^2^) = 0.120
*S* = 1.08
3108 reflections
162 parameters
2 restraints
H atoms treated by a mixture of independent and constrained refinement
Δρ_max_ = 0.28 e Å^−3^
Δρ_min_ = −0.24 e Å^−3^

### 

Data collection: *CrystalClear* (Rigaku, 2005 ▶); cell refinement: *CrystalClear*; data reduction: *CrystalClear*; program(s) used to solve structure: *SHELXS97* (Sheldrick, 2008 ▶); program(s) used to refine structure: *SHELXL97* (Sheldrick, 2008 ▶); molecular graphics: *ORTEP-3* (Farrugia, 1997 ▶) and *DIAMOND* (Brandenburg, 1998 ▶); software used to prepare material for publication: *SHELXTL* (Sheldrick, 2008 ▶).

## Supplementary Material

Crystal structure: contains datablocks I, global. DOI: 10.1107/S1600536809019084/lx2100sup1.cif

Structure factors: contains datablocks I. DOI: 10.1107/S1600536809019084/lx2100Isup2.hkl

Additional supplementary materials:  crystallographic information; 3D view; checkCIF report

## Figures and Tables

**Table 1:** Hydrogen-bond geometry (Å, °)

*D*—H⋯*A*	*D*—H	H⋯*A*	*D*⋯*A*	*D*—H⋯*A*
N1—H1⋯Cl2^i^	0.86	2.17	3.018 (2)	169
N2—H2*A*⋯Cl1^i^	0.86	2.18	3.021 (2)	166
N3—H3*A*⋯Cl2^ii^	0.90	2.20	3.058 (2)	158
N3—H3*B*⋯O1*W*	0.90	1.80	2.656 (3)	159
O1*W*—H1*A*⋯Cl1^iii^	0.85 (3)	2.22 (3)	3.069 (3)	174 (4)
O1*W*—H1*B*⋯Cl2^iv^	0.84 (3)	2.37 (4)	3.181 (2)	161 (4)

Symmetry codes: (i) 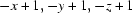; (ii) 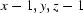; (iii) 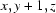; (iv) 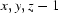.

**Fig. 1.**
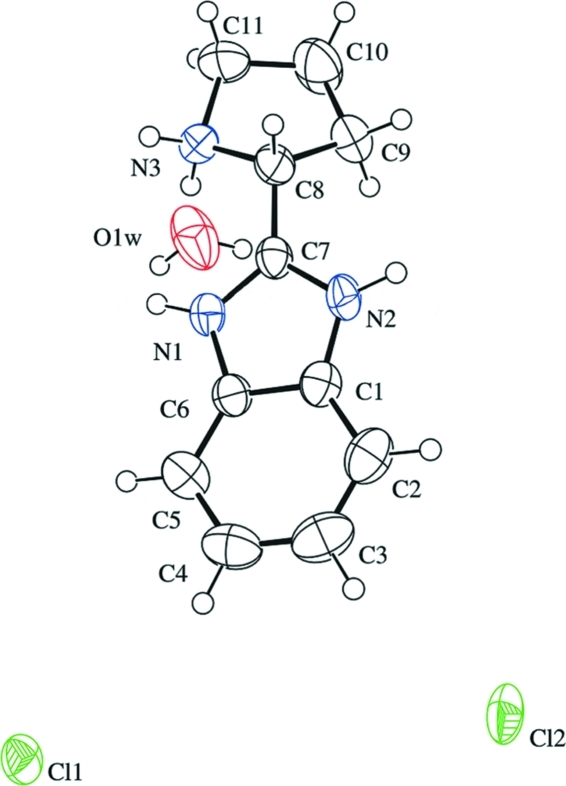
A view of the title compound with the atomic numbering scheme. Displacement ellipsoids were drawn at the 30% probability level.

**Fig. 2.**
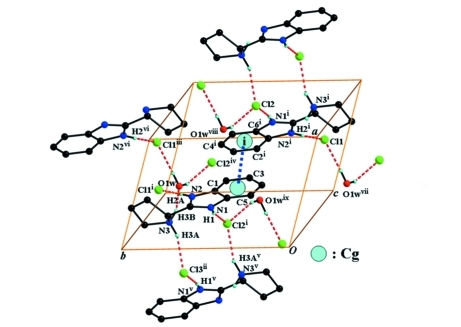
The π–π, N—H···Cl, O—H···Cl and N—H···O interactions (dotted line) in the title compound. Cg denotes the ring centroid of the C1-C6 benzene ring. [Symmetry codes: (i) -x+1, -y+1, -z+1; (ii) x-1, y, z-1; (iii) x, y+1, z; (iv) x, y, z-1; (v) -x, -y+1, -z; (vi) x+1, -y+2, -z+1; (vii) x, y-1, z; (viii) x, y, z+1; (ix) -x+1, -y+1, -z.]

## supplementary crystallographic information

### Comment

Amino acid derivatives provide wide applications in the field of material science, such as ferroelectric, fluorescence and dielectric behaviors. Also,there have been much attention in the preparation of amino acid coordination compound. (Aminabhavi *et al.*, 1986; Dai & Fu 2008*a*,*b*; Fu & Ye 2007; Fu, *et al.* 2007). Here we report the crystal structure of the title compound, (S)-2-(pyrrolidinium-2-yl)-1H-benzimidazol-3-ium dichloride monohydrate (Fig. 1).

The crystal packing (Fig. 2) is stabilized by aromatic π–π interactions between the benzene rings of the neighbouring benzimidazole systems. The Cg···Cg^i^ distance is 3.712 (2) Å (Cg is the centroide of the C1—C6 benzene ring, symmetry code as in Fig. 2). The molecular packing is further stabilized by intermolecular N—H···Cl, O—H···Cl and N—H···O hydrogen bonds (Fig. 2 and Table 1; symmetry code as in Fig. 2).

### Experimental

The homochiral ligand (S)-2-(pyrrolidin-2-yl)-1H-benzimidazole was synthesized by reaction of *S*-pyrrolidine-2-carboxylic acid and benzene-1,2-diamine according to the procedure described in the literature(Aminabhavi, *et al.*(1986)). Then (S)-2-(pyrrolidin-2-yl)-1H-benzimidazole (3 mmol) was dissolved in the solution of distilled water (20 ml) and hydrochloric acid (1 ml) and evaporated in the air affording colorless block crystals of this compound suitable for X-ray analysis.

### Refinement

All H atoms attached to C, N and O atoms were fixed geometrically and treated as riding with C—H = 0.93 Å (aromatic), 0.97 Å (methylene) or 0.98 Å (methine) and N—H = 0.90 Å (N3), 0.86 Å (N1, N2) and O—H = 0.85 Å with *U*_iso_(H) = 1.2*U*_eq_(C,*N*) and *U*_iso_(H) = 1.5*U*_eq_(O). The distances of O1W—H were restrained to 0.85 (1) Å using command DFIX.

### Crystal data

**Table:**

C_11_H_15_N_3_^2+^·2Cl^−^·H_2_O	*Z* = 2
*M**_r_* = 278.18	*F*(000) = 292
Triclinic, *P*1	*D*_x_ = 1.361 Mg m^−^^3^
Hall symbol: -P 1	Mo *K*α radiation, λ = 0.71073 Å
*a* = 7.493 (2) Å	Cell parameters from 3108 reflections
*b* = 9.739 (2) Å	θ = 3.1–27.5°
*c* = 9.937 (2) Å	µ = 0.47 mm^−^^1^
α = 99.23 (3)°	*T* = 293 K
β = 95.73 (3)°	Block, colorless
γ = 106.27 (3)°	0.35 × 0.30 × 0.15 mm
*V* = 679.0 (3) Å^3^	

### Data collection

**Table:**

Rigaku Mercury2 diffractometer	3108 independent reflections
Radiation source: fine-focus sealed tube	2310 reflections with *I* > 2σ(*I*)
graphite	*R*_int_ = 0.037
Detector resolution: 13.6612 pixels mm^-1^	θ_max_ = 27.5°, θ_min_ = 3.1°
CCD profile fitting scans	*h* = −9→9
Absorption correction: multi-scan (*CrystalClear*; Rigaku, 2005)	*k* = −12→12
*T*_min_ = 0.959, *T*_max_ = 0.982	*l* = −12→12
7119 measured reflections	

### Refinement

**Table:**

Refinement on *F*^2^	Primary atom site location: structure-invariant direct methods
Least-squares matrix: full	Secondary atom site location: difference Fourier map
*R*[*F*^2^ > 2σ(*F*^2^)] = 0.050	Hydrogen site location: difference Fourier map
*wR*(*F*^2^) = 0.120	H atoms treated by a mixture of independent and constrained refinement
*S* = 1.08	*w* = 1/[σ^2^(*F*_o_^2^) + (0.0354*P*)^2^ + 0.3615*P*] where *P* = (*F*_o_^2^ + 2*F*_c_^2^)/3
3108 reflections	(Δ/σ)_max_ < 0.001
162 parameters	Δρ_max_ = 0.28 e Å^−^^3^
2 restraints	Δρ_min_ = −0.24 e Å^−^^3^

### Special details

**Table:**

Geometry. All esds (except the esd in the dihedral angle between two l.s. planes) are estimated using the full covariance matrix. The cell esds are taken into account individually in the estimation of esds in distances, angles and torsion angles; correlations between esds in cell parameters are only used when they are defined by crystal symmetry. An approximate (isotropic) treatment of cell esds is used for estimating esds involving l.s. planes.
Refinement. Refinement of F^2^ against ALL reflections. The weighted R-factor wR and goodness of fit S are based on F^2^, conventional R-factors R are based on F, with F set to zero for negative F^2^. The threshold expression of F^2^ > 2sigma(F^2^) is used only for calculating R-factors(gt) etc. and is not relevant to the choice of reflections for refinement. R-factors based on F^2^ are statistically about twice as large as those based on F, and R- factors based on ALL data will be even larger.

### Fractional atomic coordinates and isotropic or equivalent isotropic displacement parameters (Å^2^)

**Table:**

	*x*	*y*	*z*	*U*_iso_*/*U*_eq_	
Cl1	0.81735 (10)	0.00616 (8)	0.30163 (7)	0.0600 (2)	
Cl2	0.79242 (9)	0.58762 (8)	0.97152 (7)	0.0597 (2)	
N1	0.2136 (3)	0.5855 (2)	0.31035 (18)	0.0390 (4)	
H1	0.2058	0.5445	0.2257	0.047*	
N2	0.2230 (3)	0.7465 (2)	0.48963 (18)	0.0414 (5)	
H2A	0.2224	0.8269	0.5400	0.050*	
N3	0.1820 (3)	0.7775 (2)	0.11964 (19)	0.0425 (5)	
H3A	0.0823	0.7024	0.0759	0.051*	
H3B	0.2875	0.7518	0.1123	0.051*	
C1	0.2450 (3)	0.6267 (3)	0.5381 (2)	0.0405 (5)	
C2	0.2697 (4)	0.5979 (3)	0.6704 (3)	0.0573 (7)	
H2	0.2738	0.6666	0.7483	0.069*	
C3	0.2877 (4)	0.4640 (4)	0.6804 (3)	0.0662 (8)	
H3	0.3058	0.4419	0.7674	0.079*	
C4	0.2798 (4)	0.3597 (3)	0.5650 (3)	0.0628 (8)	
H4	0.2919	0.2697	0.5766	0.075*	
C5	0.2546 (4)	0.3866 (3)	0.4338 (3)	0.0537 (6)	
H5	0.2481	0.3169	0.3562	0.064*	
C6	0.2394 (3)	0.5228 (2)	0.4234 (2)	0.0379 (5)	
C7	0.2029 (3)	0.7186 (2)	0.3528 (2)	0.0364 (5)	
C8	0.1637 (3)	0.8211 (3)	0.2668 (2)	0.0409 (5)	
H8	0.0341	0.8225	0.2709	0.049*	
C9	0.2916 (4)	0.9767 (3)	0.3080 (3)	0.0529 (6)	
H9A	0.4185	0.9793	0.3426	0.063*	
H9B	0.2454	1.0327	0.3786	0.063*	
C10	0.2868 (6)	1.0359 (3)	0.1762 (3)	0.0755 (9)	
H10A	0.2191	1.1077	0.1822	0.091*	
H10B	0.4137	1.0822	0.1609	0.091*	
C11	0.1905 (5)	0.9111 (3)	0.0618 (3)	0.0666 (8)	
H11A	0.2606	0.9135	−0.0152	0.080*	
H11B	0.0648	0.9134	0.0302	0.080*	
O1W	0.5254 (3)	0.7671 (3)	0.0856 (3)	0.0863 (8)	
H1A	0.613 (4)	0.832 (3)	0.142 (3)	0.117 (16)*	
H1B	0.572 (5)	0.707 (3)	0.043 (3)	0.102 (13)*	

### Atomic displacement parameters (Å^2^)

**Table:**

	*U*^11^	*U*^22^	*U*^33^	*U*^12^	*U*^13^	*U*^23^
Cl1	0.0704 (5)	0.0589 (4)	0.0530 (4)	0.0339 (4)	0.0092 (3)	−0.0076 (3)
Cl2	0.0536 (4)	0.0722 (5)	0.0447 (4)	0.0234 (3)	0.0033 (3)	−0.0186 (3)
N1	0.0487 (11)	0.0378 (10)	0.0303 (9)	0.0166 (9)	0.0067 (8)	−0.0010 (7)
N2	0.0501 (11)	0.0408 (11)	0.0331 (10)	0.0182 (9)	0.0101 (8)	−0.0037 (8)
N3	0.0439 (11)	0.0448 (11)	0.0382 (10)	0.0161 (9)	0.0007 (8)	0.0050 (8)
C1	0.0378 (12)	0.0459 (13)	0.0378 (12)	0.0124 (10)	0.0106 (9)	0.0056 (10)
C2	0.0588 (16)	0.0740 (19)	0.0380 (13)	0.0176 (14)	0.0137 (12)	0.0085 (13)
C3	0.0662 (19)	0.086 (2)	0.0546 (17)	0.0220 (17)	0.0159 (14)	0.0355 (16)
C4	0.0642 (18)	0.0557 (17)	0.077 (2)	0.0197 (14)	0.0146 (15)	0.0316 (15)
C5	0.0593 (16)	0.0436 (14)	0.0600 (17)	0.0172 (12)	0.0119 (13)	0.0104 (12)
C6	0.0380 (12)	0.0374 (12)	0.0381 (12)	0.0111 (10)	0.0082 (9)	0.0052 (9)
C7	0.0357 (11)	0.0376 (12)	0.0350 (11)	0.0123 (9)	0.0076 (9)	0.0010 (9)
C8	0.0392 (12)	0.0445 (13)	0.0420 (13)	0.0197 (10)	0.0086 (10)	0.0031 (10)
C9	0.0599 (16)	0.0413 (14)	0.0550 (16)	0.0156 (12)	0.0086 (13)	0.0021 (11)
C10	0.110 (3)	0.0456 (16)	0.069 (2)	0.0160 (17)	0.0164 (18)	0.0150 (14)
C11	0.092 (2)	0.0606 (18)	0.0564 (18)	0.0338 (17)	0.0045 (16)	0.0239 (14)
O1W	0.0539 (13)	0.0682 (15)	0.125 (2)	0.0221 (12)	0.0145 (14)	−0.0227 (14)

### Geometric parameters (Å, °)

**Table:**

N1—C7	1.324 (3)	C4—H4	0.9300
N1—C6	1.384 (3)	C5—C6	1.381 (3)
N1—H1	0.8600	C5—H5	0.9300
N2—C7	1.327 (3)	C7—C8	1.484 (3)
N2—C1	1.376 (3)	C8—C9	1.514 (3)
N2—H2A	0.8600	C8—H8	0.9800
N3—C8	1.487 (3)	C9—C10	1.514 (4)
N3—C11	1.492 (3)	C9—H9A	0.9700
N3—H3A	0.9000	C9—H9B	0.9700
N3—H3B	0.9000	C10—C11	1.481 (4)
C1—C6	1.386 (3)	C10—H10A	0.9700
C1—C2	1.393 (3)	C10—H10B	0.9700
C2—C3	1.366 (4)	C11—H11A	0.9700
C2—H2	0.9300	C11—H11B	0.9700
C3—C4	1.389 (4)	O1W—H1A	0.85 (3)
C3—H3	0.9300	O1W—H1B	0.84 (3)
C4—C5	1.375 (4)		
			
C7—N1—C6	109.38 (18)	N1—C6—C1	105.90 (19)
C7—N1—H1	125.3	N1—C7—N2	108.91 (19)
C6—N1—H1	125.3	N1—C7—C8	127.69 (19)
C7—N2—C1	109.25 (18)	N2—C7—C8	123.30 (19)
C7—N2—H2A	125.4	C7—C8—N3	112.88 (18)
C1—N2—H2A	125.4	C7—C8—C9	115.7 (2)
C8—N3—C11	104.06 (19)	N3—C8—C9	103.78 (19)
C8—N3—H3A	110.9	C7—C8—H8	108.1
C11—N3—H3A	110.9	N3—C8—H8	108.1
C8—N3—H3B	110.9	C9—C8—H8	108.1
C11—N3—H3B	110.9	C8—C9—C10	104.4 (2)
H3A—N3—H3B	109.0	C8—C9—H9A	110.9
N2—C1—C6	106.55 (19)	C10—C9—H9A	110.9
N2—C1—C2	132.8 (2)	C8—C9—H9B	110.9
C6—C1—C2	120.7 (2)	C10—C9—H9B	110.9
C3—C2—C1	116.9 (3)	H9A—C9—H9B	108.9
C3—C2—H2	121.6	C11—C10—C9	107.4 (2)
C1—C2—H2	121.6	C11—C10—H10A	110.2
C2—C3—C4	122.2 (3)	C9—C10—H10A	110.2
C2—C3—H3	118.9	C11—C10—H10B	110.2
C4—C3—H3	118.9	C9—C10—H10B	110.2
C5—C4—C3	121.5 (3)	H10A—C10—H10B	108.5
C5—C4—H4	119.3	C10—C11—N3	105.7 (2)
C3—C4—H4	119.3	C10—C11—H11A	110.6
C4—C5—C6	116.5 (3)	N3—C11—H11A	110.6
C4—C5—H5	121.8	C10—C11—H11B	110.6
C6—C5—H5	121.8	N3—C11—H11B	110.6
C5—C6—N1	131.8 (2)	H11A—C11—H11B	108.7
C5—C6—C1	122.3 (2)	H1A—O1W—H1B	109 (4)
			
C7—N2—C1—C6	0.6 (2)	C6—N1—C7—N2	0.8 (2)
C7—N2—C1—C2	−179.6 (3)	C6—N1—C7—C8	−175.7 (2)
N2—C1—C2—C3	−179.8 (3)	C1—N2—C7—N1	−0.9 (3)
C6—C1—C2—C3	0.0 (4)	C1—N2—C7—C8	175.8 (2)
C1—C2—C3—C4	−0.7 (4)	N1—C7—C8—N3	−14.5 (3)
C2—C3—C4—C5	0.4 (5)	N2—C7—C8—N3	169.4 (2)
C3—C4—C5—C6	0.6 (4)	N1—C7—C8—C9	−133.8 (2)
C4—C5—C6—N1	−180.0 (2)	N2—C7—C8—C9	50.1 (3)
C4—C5—C6—C1	−1.3 (4)	C11—N3—C8—C7	−164.9 (2)
C7—N1—C6—C5	178.4 (3)	C11—N3—C8—C9	−38.9 (2)
C7—N1—C6—C1	−0.4 (2)	C7—C8—C9—C10	154.4 (2)
N2—C1—C6—C5	−179.1 (2)	N3—C8—C9—C10	30.1 (3)
C2—C1—C6—C5	1.1 (4)	C8—C9—C10—C11	−10.2 (3)
N2—C1—C6—N1	−0.1 (2)	C9—C10—C11—N3	−13.5 (4)
C2—C1—C6—N1	−180.0 (2)	C8—N3—C11—C10	32.6 (3)

### Hydrogen-bond geometry (Å, °)

**Table:**

*D*—H···*A*	*D*—H	H···*A*	*D*···*A*	*D*—H···*A*
N1—H1···Cl2^i^	0.86	2.17	3.018 (2)	169
N2—H2A···Cl1^i^	0.86	2.18	3.021 (2)	166
N3—H3A···Cl2^ii^	0.90	2.20	3.058 (2)	158
N3—H3B···O1W	0.90	1.80	2.656 (3)	159
O1W—H1A···Cl1^iii^	0.85 (3)	2.22 (3)	3.069 (3)	174 (4)
O1W—H1B···Cl2^iv^	0.84 (3)	2.37 (4)	3.181 (2)	161 (4)

Symmetry codes: (i) −*x*+1, −*y*+1, −*z*+1; (ii) *x*−1, *y*, *z*−1; (iii) *x*, *y*+1, *z*; (iv) *x*, *y*, *z*−1.
